# Augmented Features Synergize Radiomics in Post-Operative Survival Prediction and Adjuvant Therapy Recommendation for Non-Small Cell Lung Cancer

**DOI:** 10.3389/fonc.2022.659096

**Published:** 2022-01-31

**Authors:** Lawrence Wing-Chi Chan, Tong Ding, Huiling Shao, Mohan Huang, William Fuk-Yuen Hui, William Chi-Shing Cho, Sze-Chuen Cesar Wong, Ka Wai Tong, Keith Wan-Hang Chiu, Luyu Huang, Haiyu Zhou

**Affiliations:** ^1^ Department of Health Technology and Informatics, The Hong Kong Polytechnic University, Hong Kong, Hong Kong SAR, China; ^2^ Department of Clinical Oncology, Queen Elizabeth Hospital, Hong Kong, Hong Kong SAR, China; ^3^ Department of Diagnostic Radiology, University of Hong Kong, Hong Kong, Hong Kong SAR, China; ^4^ Department of Thoracic Surgery, Guangdong Provincial People’s Hospital, Guangdong Academy of Medical Sciences, School of Medicine, South China University of Technology, The Second School of Clinical Medicine, Southern Medical University, Guangzhou, China; ^5^ Department of Thoracic Surgery, Jiangxi Lung Cancer Institute, Jiangxi Cancer Hospital, Nanchang, China

**Keywords:** non-small cell lung cancer (NSCLC), radiomics, adjuvant therapy (post-operative), prediction model, patient benefit

## Abstract

**Background:**

Owing to the cytotoxic effect, it is challenging for clinicians to decide whether post-operative adjuvant therapy is appropriate for a non-small cell lung cancer (NSCLC) patient. Radiomics has proven its promising ability in predicting survival but research on its actionable model, particularly for supporting the decision of adjuvant therapy, is limited.

**Methods:**

Pre-operative contrast-enhanced CT images of 123 NSCLC cases were collected, including 76, 13, 16, and 18 cases from R01 and AMC cohorts of The Cancer Imaging Archive (TCIA), Jiangxi Cancer Hospital and Guangdong Provincial People’s Hospital respectively. From each tumor region, 851 radiomic features were extracted and two augmented features were derived therewith to estimate the likelihood of adjuvant therapy. Both Cox regression and machine learning models with the selected main and interaction effects of 853 features were trained using 76 cases from R01 cohort, and their test performances on survival prediction were compared using 47 cases from the AMC cohort and two hospitals. For those cases where adjuvant therapy was unnecessary, recommendations on adjuvant therapy were made again by the outperforming model and compared with those by IBM Watson for Oncology (WFO).

**Results:**

The Cox model outperformed the machine learning model in predicting survival on the test set (C-Index: 0.765 vs. 0.675). The Cox model consists of 5 predictors, interestingly 4 of which are interactions with augmented features facilitating the modulation of adjuvant therapy option. While WFO recommended no adjuvant therapy for only 13.6% of cases that received unnecessary adjuvant therapy, the same recommendations by the identified Cox model were extended to 54.5% of cases (McNemar’s test *p* = 0.0003).

**Conclusions:**

A Cox model with radiomic and augmented features could predict survival accurately and support the decision of adjuvant therapy for bettering the benefit of NSCLC patients.

## 1 Background

Lung cancer is the leading cause of cancer-related deaths worldwide, in which non-small cell lung cancer (NSCLC) is the most common primary lung malignancy cancer, accounting for 85% of all cases (1). For early-stage NSCLC (IA-IIB) (2), and some locally advanced NSCLC (stage IIIA) (3), complete surgical resection is considered as the most optimal treatment option. Combined modality therapy, including chemotherapy, radiation therapy and surgery, could be applied for NSCLC that cannot be completely removed by surgery (1). Despite the advancement in treatment, the recurrence rate following complete surgical resection of NSCLC tumor remains high, ranging from 30% to 75% (3). In order to reduce the recurrence rate and prolong the patient’s survival, there are two countermeasures that could be applied on the patients: pre-operative induction therapy and post-operative adjuvant therapy where their net benefits are similar. Post-operative adjuvant therapy is regarded as the better option because the clinicians can assess the patients’ pre-operative conditions for adjuvant therapy. Post-operative thoracic radiotherapy can reduce the chance of recurrence after surgery, but some previous studies revealed its detrimental effect on the patient’s survival (4). Although platinum-based chemotherapy is more suitable for stage IB, II, and III NSCLC patients, some patients could not tolerate its cytotoxic effect (4, 5). Therefore, it is necessary for clinicians to consider the survival benefits and risks of adjuvant therapy on patients.

In reality, the conventional Tumor Node Metastasis (TNM) staging system, which is the most important post-operative prognostic factor, may not be capable to stratify the recurrence risk of patients accurately. It was revealed that the recurrence and the survival time of NSCLC patients in the same TNM stage were diverse (6), and even the recurrence rate of stage IA NSCLC is still high, reaching 30% (5). Therefore, in the era of precision medicine, a quantitative predictive model is required to evaluate the recurrence risk of NSCLC patients and the effect of adjuvant therapy on survival.

Radiomics is a newly emerged field in radiology that involves the high-throughput extraction of quantitative features from the regions of interest in medical images, which allows the use of non-invasive imaging technique for monitoring tumor progression and treatment outcome. Previously, there has been substantial interest in relating the radiomic features with the prognosis and pathological response to treatments of NSCLC patients, which related radiomic features with patients’ survival (2, 7–9). However, the role of adjuvant therapy was not taken into account in these studies, and it remains unclear whether their findings could be used to evaluate the survival benefit of post-operative adjuvant therapy.

This work proposed a quantitative radiomics model that involves adjuvant-therapy-associated features for predicting the patient’s survival. The proposed model can provide the clinicians with an additional diagnostic tool to determine whether post-operative adjuvant therapy is appropriate for NSCLC patients.

## 2 Methods

### 2.1 Data Collection and Radiomic Feature Extraction

This is a retrospective study on the anonymized pre-operative contrast-enhanced CT images together with the clinical information of 123 NSCLC cases that were collected, including 76, 13, 16, and 18 cases from R01 and AMC cohorts of The Cancer Imaging Archive (TCIA), Jiangxi Cancer Hospital and Guangdong Provincial People’s Hospital, respectively. For the open access image data, the CT scanning parameters are a slice thickness of 0.625–3 mm, a pixel spacing of 0.604–0.930, and an x-ray tube current of 124–699 mA at 80–140 kVp. For the image data collected from Jiangxi Cancer Hospital, the CT scanning parameters are a slice thickness of 1 mm, a pixel spacing of 0.781, and an X-ray tube current of 287 mA at 140 kVp. For the image data collected from Guangdong Provincial People’s Hospital, the CT scanning parameters are a slice thickness of 1 mm, a pixel spacing of 0.742, and an x-ray tube current of 378 mA at 120 kVp.

The open access data originally consist of 211 NSCLC cases of the R01 and AMC cohorts, which were collected between April 2008 and September 2012, from TCIA database (10–12). Institutional review board approval was waived as the patient data in TCIA have been deidentified. All the cases have undergone surgical resection of the tumor with preoperative CT performed prior to surgical procedures. Among the 211 cases, tumor segmentation was performed in 76 cases from the R01 cohort, which were used as the training set. As the segmentations were unavailable in the AMC cohort, 13 cases with clear semantic annotations of tumor manifestations were included in the test set. The test set consists of 47 cases from the AMC cohort and two hospitals. The contouring of tumors for the test set was performed by WH and KT and validated by KC based on the semantic annotations provided. The summary of the patients is shown in **Table 1**.

**Table 1:** Summary of the study cohorts. Jiangxi: Jiangxi Cancer Hospital; Guangdong: Guangdong Provincial People’s Hospital.

	Training set		Test set	
Cohort	R01	AMC	Jiangxi	Guangdong
Sample size	76	13	16	18
Gender				
Male	57	3	5	8
Female	19	10	11	10
Age (years)	70 (43–87)	69 (40–80)	58.5 (40–71)	59 (39–74)
Adjuvant therapy				
Chemotherapy only	15	2	16	18
Combined chemotherapy/radiotherapy	7	0	0	0
Overall survival (days)	1286 (20–3175)	874 (447–1341)	950 (395–1429)	758 (212–1186)

The radiomic features were extracted from the images using Slicer Radiomics with a fixed bin width of 25 and following the definitions provided by Image Biomarker Standardization Initiative (IBSI) (13). A total of 851 radiomic features, composed of 14 shape features, 18 intensity features, 75 texture features, and 744 wavelet features, were extracted from the regions of interest. Laplacian of Gaussian wavelet was applied to the images before extracting the wavelet features. The default bin width, 25, was applied to all texture features.

### 2.2 Computation of Concordance Index

Concordance Index (C-Index) was used to quantify the performance of model, which was computed based on the following area under the curve (AUC) formula (14).


(1) $$AUC(\tau )=P(P_{i}>P_{j}|T_{i}^{S}<T_{j}^{S},T_{i}^{S}<\tau )$$

where τ represents the observe period; P_i_ and P_j_ represent the relative hazard any two patients experience; ${\text{TiS}}_{\text{ and T}}^{\text{jS}}$ represent their survival time. The detailed procedure for computing C-Index is illustrated in **Supplementary Figure 1**.

### 2.3 Derivation of Augmented Features

Augmented features were derived based on Pearson’s correlation coefficients between primary predictors (15), which are the radiomic features in this work. As the primary predictors should follow the normal distribution, rank-based inverse normalization was performed to the radiomic features. The association level between two radiomic features, z_i_ and z_j_, was denoted by C_T_(i, j) for the adjuvant therapy group of n_1_ cases and C_N_(i, j) for the control group of n_0_ cases without adjuvant therapy and is quantified by the following formulas.


(2) $$C_{T}(i,j)=\left|\frac{1}{n_{1}}\Sigma_{h=1}^{n_{1}}\;z_{i}(h)z_{j}(h)\right|$$

(3) $$C_{N}(i,j)=\left|\frac{1}{n_{0}}\Sigma_{h=1}^{n_{0}}\;z_{i}(h)z_{j}(h)\right|$$

Two cumulative distributions, F_T_ and F_N_, formed by C_T_ and C_N_, represent the association structures among radiomic features in the adjuvant therapy and control groups, which were compared using two-sample Kolmogorov–Smirnov test (16). With significant difference, the likelihood of adjuvant therapy can be estimated by two augmented features given by the following formulas (15).


(4) $$z\prime_{1}(h)={\left(\Sigma_{i=1}^{m}z_{i}(h)\right)}^{2}$$

(5) $$z\prime_{2}(h)={\left(\Sigma_{i=1}^{m}|z_{i}(h)|\right)}^{2}$$

### 2.4 Model Identification

**Figure 1:**
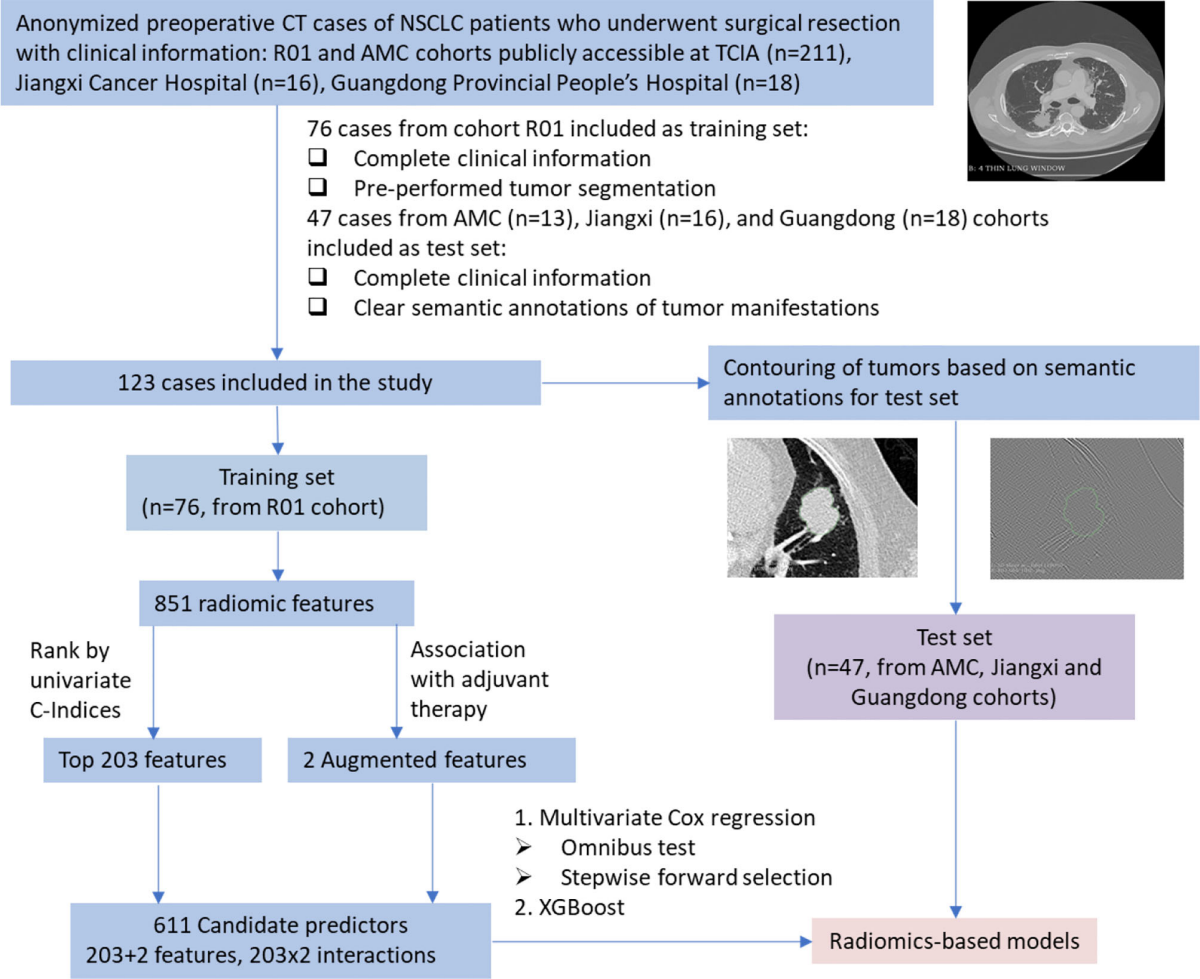
Flow chart of model identification and performance test. NSCLC, non-small cell lung cancer; CT, computed tomography; TCIA, The Cancer Imaging Archive; Jiangxi, Jiangxi Cancer Hospital; Guangdong, Guangdong Provincial People’s Hospital.

In survival data, the survival time is defined as the days between dates first CT diagnosis and last known survival and the status indicates whether the time is right-censored. A linear Cox model and a machine learning model were built for comparison. For the linear model, the radiomic features were first evaluated by the rank of C-indices of the 851 univariate Cox models, where the top 203 features were selected as candidates for the multivariate Cox regression model. To examine the effect of adjuvant therapy on survival, the augmented features were included as interactions with the selected 203 radiomic features, which gave 611 candidate predictors in total. Feature selection was further performed on the 611 predictors based on the omnibus test with entry and removal criteria at 0.05 and 0.10 respectively. The nomogram was generated using the coefficients of Cox regression, and the model fit was assessed by calibration plot. The flow chart of the model identification and performance test is illustrated in **Figure 1**. Setting the predicted outcome as proportional hazard, a machine learning model of decision trees was fitted using XGBoost (17).

### 2.5 Statistical Analysis

A risk score was defined as the log relative hazard of each patient, which was used to stratify the cases into high- and low-risk groups by the median risk scores. To examine the discriminative ability of the risk score, a two-sample log rank test was performed to the survival functions of the two groups estimated by the Kaplan–Meier estimator.

The distribution of C-Index under null hypothesis was simulated by the Monte Carlo procedure. While the panel of selected predictors were kept unchanged, the survival time together with the event status were randomly permutated 1,000 times. Using the same predictors, 1,000 null models were generated, whose C-indices were used to simulate the null distribution. Statistical significance quantified by *p*-value was obtained by the area under the simulated null distribution at the right tail delimited by the C-Index of the identified model.

The statistical analyses were performed by the statistical package (SPSS version 24.0), and the packages survival, rms, and xgboost in the R statistical software (version 3.6.3; www.r-project.org). *p*-values less than 0.05 were considered as significant.

### 2.6 Derivation of Clinical Suggestion on Adjuvant Therapy

After the predictive model has been obtained, clinical suggestion could be given by first obtaining the radiomics data of the patients and treating the two augmented features $z\prime_{1}\text{ and }z\prime_{2}$ as the actionable variables. For clinicians to make clinical decisions for a new case, the risk score is inferred by inputting the radiomics data extracted from the CT images and modulating the value of $z\prime_{1}\text{ and }z\prime_{2}$ for optimizing the survival benefit. In other words, the log relative hazard of the patient could be seen as a function of $z\prime_{1}\text{ and }z\prime_{2}$ . The following formula deduces the values of $z\prime_{1}\text{ and }z\prime_{2}$ that favor the longest survival, i.e., lowest relative risk.


(6) $$\left(v_{1},v_{2})=\underset{z\prime_{1},z\prime_{2}}{\arg\min}y(z\prime_{1},z\prime_{2}\right)$$

where y $\left(z\prime_{1},z\prime_{2}\right)$ represents the Cox model with fixed radiomic features. The values of $z\prime_{1}\text{ and }z\prime_{2}$ are both between 0 and 1 and their geometric mean, G_T_, also falls on the same range. This work also proved that G_T_ is inversely associated with the likelihood of adjuvant therapy. Thus, G_T_ = 0 corresponds to adjuvant therapy and G_T_ = 1 corresponds to no adjuvant therapy. Due to the property of geometric mean, the same implication applies to $z\prime_{1}\text{ and }z\prime_{2}$ . To simulate the outcome with and without adjuvant therapy, we could simply set $z\prime_{1}\text{ and }z\prime_{2}$ to be both 0 and both 1, respectively, to determine which pair gives a lower risk, i.e., (*v*_1_, *v*_2_).

### 2.7 Determination of Patients’ Benefit From the Best Model

Given the toxicity and side effects of adjuvant therapy, the net benefit from clinical decision is defined by whether adjuvant therapy is necessary for the patients. To do this, we defined a group of patients who did not receive adjuvant therapy, and recurrence also did not happen, implying unnecessity of adjuvant therapy.

To compare the benefits of the identified model and the actual clinical decision made by clinicians, medical information of patients, including pathologic data, such as TNM stage, was input into IBM’s Watson for Oncology (WFO) (https://www.ibm.com/watson-health/oncology-and-genomics), where the actual decision is regarded as reference. We excluded 6 patients who had local recurrence since reference data of the treatment is currently not available in WFO. We compared the proportion of better decisions made by the identified model and WFO to decide whether the model is more beneficial to the patients.

However, it is inappropriate to define a group of patients who received adjuvant therapy, and recurrence also did not happen, that could indicate the necessity of adjuvant therapy. It is because the absence of recurrence was not necessarily caused by adjuvant therapy.

## 3 Results

### 3.1 Model Comparison and Predictive Ability of Radiomic Features

The C-Index of the linear model was 0.7834 for the training data and 0.765 for the test data. It is worth noting that the test performance was not lowered by our own tumor segmentations. However, while the C-Index of the XGBoost machine learning model was 0.841 for the training data, that for the test data was only 0.675, indicating poorer performance. The trained XGBoost tree model is shown in **Supplementary Figure 2**. Therefore, the linear Cox model was chosen for further analysis.

When building the linear model, among the top 203 features, C-Index ranged from 0.5647 to 0.6197. The top 203 instead of the top 200 were selected because the 199th–203rd features gave the same C-Index value. The performance of radiomic features is summarized in **Table 2** with respect to the four categories.

**Table 2:** Predictive ability of radiomic features in four categories.

Category	Total number of features in the category	Number of top 203 features in the category	The best-performing feature in the category	C-Index of the best performing feature
Shape	14	2	Surface Area	0.5690
Intensity	18	3	Energy	0.5776
Texture	75	22	Large Area Low Gray Level Emphasis	0.6069
Wavelet	744	176	HLL-Energy	0.6197

### 3.2 Association of Augmented Features With Adjuvant Therapy

**Figure 2:**
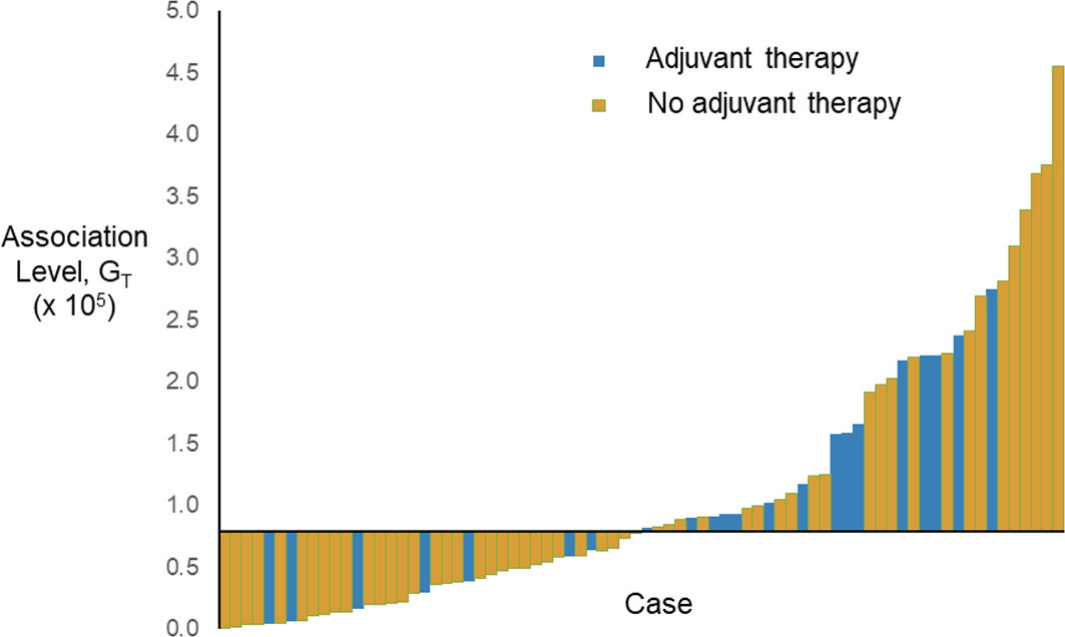
Waterfall plot of the estimated association level, G_T_, sorted across the cases (before rescaled). The horizontal axis crosses the vertical axis at the median of G_T_, i.e., 0.796 × 10^5^.

As the augmented features, z’_1_ and z’_2_, represent the second-order statistics of radiomic features as shown in Equations (4) and (5), the expected level of association with adjuvant therapy can be estimated by the geometric mean, G_T_ = (z’_1_×z’_2_)^1/2^. The waterfall plot of the estimated association level, G_T_, sorted across the cases (before rescaled) is shown in **Figure 2**. The median-dichotomized G_T_ is significantly and inversely associated with adjuvant therapy (Chi-square = 4.094, *p* = 0.043; Ordinal by Ordinal Spearman’s ρ = −0.232, *p* = 0.044).

### 3.3 Model Development and Validation

Statistical analysis selected 5 predictors: z_47_, z’_1_×z_2_, z’_1_×z_61_, z’_1_×z_92_, and z’_2_×z_175_, which are summarized in **Table 3**. The model is represented by the following equation.


(7) $$\text{y}=0.940\;z_{47}-1.957\;z\prime_{1}\times z_{2}+3.981\;z\prime_{1}\times z_{61}-2.373\;z\prime_{1}\times z_{92}-2.122\;z\prime_{2}\times z_{175}$$

**Table 3:** Summary of predictors selected for multivariate Cox regression model.

Predictor name	Main/interaction effect	Category	Radiomic feature name	*B*	*p*
wavelet-HHH_glcm_Imc1	Main effect	wavelet-HHH	Average of informational measure of correlation 1	0.940	0.003
z’_1_ × wavelet-HHL_firstorder_Skewness	Interaction with z’_1_	wavelet-HHL	Skewness	−1.951	0.003
z’_1_ × original_glrlm_ShortRun HighGrayLevelEmphasis	Interaction with z’_1_	Texture	Short-Run High Gray-level Emphasis	3.981	0.000
z’_1_ × wavelet-LHH_firstorder_Mean	Interaction with z’_1_	wavelet-LHH	Mean	−2.373	0.001
z’_2_ × wavelet-LHH_glcm_Imc1	Interaction with z’_2_	wavelet-LHH	Average of informational measure of correlation 1	−2.122	0.014

where y is log of relative hazard, h(t)/h_0_(t); h(t) represents the expected hazard at time t and h_0_(t) is the baseline hazard. The correlation coefficients of all pairs of selected covariates were calculated. The range of correlation coefficients is −0.291 to 0.512, implying that the collinearity between the model covariates is very low. The nomogram of the model is shown in **Figure 3A**.

**Figure 3:**
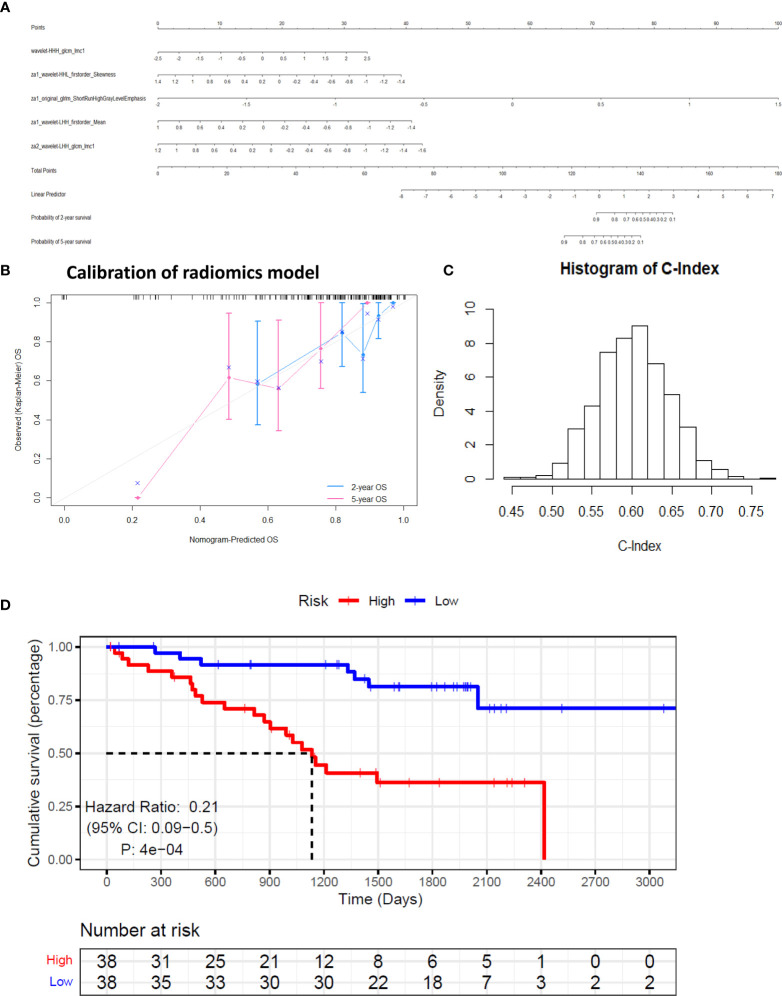
Calibration and performance of the identified model based on training set. **(A)** The identified model was illustrated as a nomogram to predict overall survival (OS). **(B)** Calibration of the model. **(C)** Simulated null distribution of C-Index. **(D)** Kaplan–Meier estimates of the survival functions of the high- and low-risk groups.

The augmented features representing the summaries of all the 851 radiomic features help stabilize the predictor values, which may be perturbed by the variation in manual tumor segmentations. The predicted overall survival (OS) was well calibrated with the Kaplan-Meier estimated OS at 2 and 5 years (**Figure 3B**). The simulated null distribution of the C-Index is shown in **Figure 3C**, which centered at 0.602, with a standard deviation of 0.0444. The highest simulated C-Index, 0.7684, is smaller than the C-Index of the identified model, 0.7834. Based on the right tail of the null distribution, the *p*-value of the identified model is empirically zero. Kaplan–Meier estimates of survival functions of the high- and low-risk groups are illustrated in **Figure 3D** (log-rank test, *p* < 0.01). Clearly from the result, individuals in the low-risk group have significantly better survival.

In addition, the simulation showed that the random performance may not be C-Index = 0.5, which was frequently assumed by previous studies (18). The criteria for significant performance may be more stringent as the null distribution was shifted to the right based on our observation.

### 3.4 Indication on Clinical Decision

**Figure 4:**
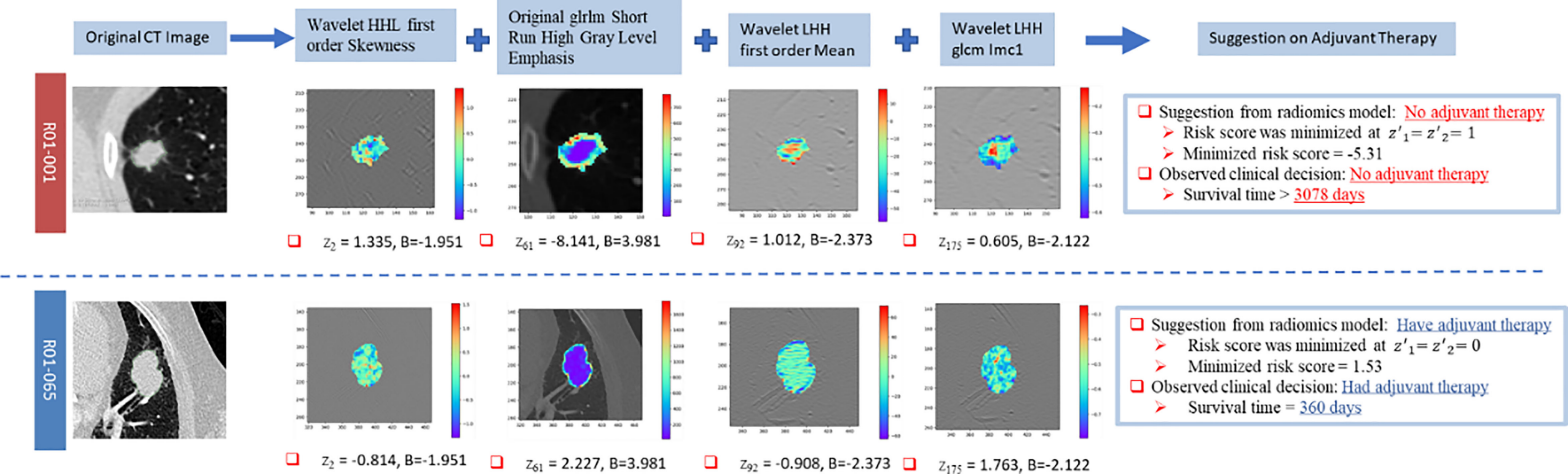
Illustration of the clinical suggestions on adjuvant therapy by two representative cases based on the identified model. “B” represents the coefficient of the corresponding covariate in the Cox model.

The suggestion on adjuvant therapy was given by minimizing the log relative hazard in Equation 7. Two representative cases, R01-001 and R01-065, were compared in **Figure 4** as an illustration of the clinical suggestions on adjuvant therapy. It was clearly shown in these two cases that the predicted risk scores accorded with the survival time and the suggestions on adjuvant therapy complied with the original decisions.

Comparison between the adjuvant therapy options suggested by the identified radiomic Cox model and WFO is shown in **Table 4**. Whereas only 13.6% of clinical decisions given by WFO were beneficial to the patients, the radiomic Cox model gave 52.0% better clinical suggestions (McNemar’s test χ^2^ = 13.136; *p* = 0.0003).

**Table 4:** Comparison between radiomic Cox model and WFO on patient’s benefit.

	Adjuvant therapy suggestion	Radiomic Cox model		Total
		Yes	No	
WFO	Yes	18	20	38
	No	2	4	6
	Total	20	24	44

## 4 Discussion

The present study proposed a predictive model based on radiomic features associated with adjuvant therapy. It achieved a high prediction accuracy, where the prediction power was proved to be statistically significant. The model could serve as an additional tool to help the clinicians know more about the survival benefits of adjuvant therapy for a NSCLC patient.

The identified model consists of 5 radiomic features. Wavelet filters were applied before the extraction of 3 radiomic features: Average of informational measure of correlation 1 (IMC1), Skewness, and Mean. A texture feature without filtering, Short-Run High Gray-level Emphasis (SRHGLE), was also selected. SRHGLE quantifies the distribution of the short homogeneous runs with low or high gray levels. It was found previously that SRHGLE was significantly associated with progression-free survival where hazard ratio = 2.43 (*p* = 0.005) concurs with the positive coefficient obtained in this work (19). IMC1 assesses the correlation between the probability distributions of two pixels separated by 1-pixel distance that quantifies the complexity of the texture based on mutual information. Tumor spatial heterogeneity was characterized by IMC1, whose exponential increase was associated with shorter overall survival in NSCLC (20). The extraction of IMC1 was performed after applying wavelet-HHH and wavelet-LHH in this work. In another study, multivariate regression analysis of radiomic features extracted from baseline CT showed that skewness of positive pixel values was associated with overall survival (21). Mean is a typical radiomic feature for classifying the tumor histopathology of NSCLC (22).

It was observed from the top 203 features and selected predictors that wavelet features have superior performance than other features in terms of C-Index and the high-frequency filters could enhance the characterization of the underlying physiopathology and anatomy of NSCLC tumor.

In this study, the performance of the Cox model and the machine learning model in predicting survival on the test set attained C-indices of 0.765 and 0.675, respectively. In contrast, Aerts et al. developed a similar Cox model based on radiomic features where the highest C-Index attained was 0.69 only (18).

The selected radiomic features, wavelet-LHH_firstorder_Mean and wavelet-LHH_glcm_Imc1, are classified in the wavelet category, which were computed after applying the wavelet-LHH filter onto the CT images. The wavelet-LHH filter applies low-pass filter on the x-dimension and high-pass filter on the y- and z-dimensions of CT images. For another selected predictor, wavelet-HHH filter was also applied before extracting IMC1. The high-frequency filter can enhance the tumor spatial heterogeneity to be quantified by IMC1 and thus the determination of tumor solid components. It is consistent with a previous study on lung cancer that solid components were regarded as invasive (23). A similar effect of high-frequency filter, wavelet-HHL, was found on skewness as a similar negative coefficient was estimated.

In the identified model, the relative hazard was related to not only the radiomic features, but also two augmented features, which reflect the likelihood of adjuvant therapy due to the significant association with their geometric mean, G_T_ (*p* = 0.043). The inverse association of augmented features with adjuvant therapy implies that the clinical decision less likely supports adjuvant therapy when the augmented features tend to 1 and more likely supports adjuvant therapy when the augmented features tend to 0. With the identified model, two survival functions can be derived by setting the augmented features to 0 for adjuvant therapy and 1 for no adjuvant therapy, respectively. Before surgery, patient’s survival durations and benefits with and without adjuvant therapy can be simulated by the two survival functions using Harrell’s approach (24).

From the European Society for Medical Oncology (ESMO) guideline, it has become clear in the past two decades that adjuvant chemotherapy benefits stage II and stage III NSCLC by improving 4%–5% absolute survival at 5 years (25). However, adjuvant chemotherapy is not recommended for stage IA and its benefit in stage IB is small based on lower evidence (26). However, the proposed model could enrich the ESMO guideline to predict the benefit of adjuvant therapy option in NSCLC at any stage.

While Watson for Oncology made decisions referring to the NCCN guideline and clinicians in Memorial Sloan Kettering based mainly on clinical information including pathological stages and ages, our model was identified based on the pre-operative CT. The significant benefit on the decision of adjuvant therapy of our model over Watson for Oncology shows the power of pre-operative CT imaging in predicting tumor progression and indicating treatment plans. In clinical practice, the poor tumor response to adjuvant therapy predicted by clinical and histopathological factors suggests a “watch and wait” approach. To provide more prognostically informative indicators for those predicted non-responders, researchers proposed to grade the colorectal cancer by counting the poorly differentiated clusters (PDCs) of neoplastic cells in histological samples and pre-operative biopsies (27, 28). A significant association between PDC grade and survival was found. The finding of this study reveals the potential of the Cox model with radiomic features in complementing the PDC grading system for the best survival benefit in NSCLC.

A similar research study was performed to develop and validate a quantitative radiomic risk score (QuRiS) for stratifying the early-stage NSCLC patients into high-, intermediate-, and low-risk groups, and the associated nomogram (QuRNom) for estimating the survival benefit (29). It was found in the high-risk group identified by QuRiS that the survival time of patients received adjuvant chemotherapy after surgery was significantly longer than patients who underwent surgery alone. The survival benefit estimated by QuRNom can predict the improvement of survival time by adjuvant chemotherapy. However, the report benefit was focused on a group of patients but not individuals. The variation of features among individuals within the same risk group cannot be addressed by such approach. In contrast, this study proposed and developed a theranostic model that can quantify the change in survival or relapse time due to the treatment and indicate the individual’s benefit.

The interobserver variability of the cases was unknown as it requires contouring. In addition, combination of clinical features was not performed in our model.

As more high-throughput radiomics data become accessible and available, personalized medicine through radiomics can help in the clinical treatment and prolong the patients’ survival case by case. The current study proposed a survival model that can predict the survival benefits of adjuvant therapy in NSCLC without reliance on stage, which can help in the clinical decisions. The model building process can also help future studies in dealing with high-dimensional data analysis.

## Data Availability Statement

Publicly available datasets were analyzed in this study. These data can be found here: https://wiki.cancerimagingarchive.net/display/Public/NSCLC+Radiogenomics.

## Ethics Statement

As the publicly available data have been anonymized, institutional review board approval was waived. Institutional review board approval was obtained from Research Ethics Committee, Jiangxi Cancer Hospital (2021ky039) and Research Ethics Committee, Guangdong Provincial People’s Hospital, Guangdong Academy of Medical Sciences (GDREC2020233H(R1)).

## Author Contributions

LC: Original draft, conceptualization, funding acquisition, project coordination, supervision, writing, review & editing. TD: Methodology, data curation, visualization, writing, review & editing. HS: Methodology & visualization. MH: review & editing. WH: Original draft, conceptualization. WC: Supervision, project administration, methodology, writing, review & editing. S-CW: Methodology, review & editing. KT: review & editing. KC: Methodology, review & editing. LH: Methodology, review & editing. HZ: Funding acquisition, project coordination, supervision, writing, review & editing. All authors contributed to the article and approved the submitted version.

## Funding

This study was funded by two Health and Medical Research Funds (HMRF 02131026 and HMRF 16172561) of Food and Health Bureau, Hong Kong; Huawei Technologies Co. Ltd. Collaborative Research Fund 2021 (PolyU Ref.: ZGBH); the Guangdong Province Medical Scientific Research Foundation (Grant Number B2018148); Science and Technology Program of Guangzhou (Grant Number 201903010028); and Natural Science Foundation of Guangdong (Grant Number 2018A0303130113). The funder was not involved in the study design, collection, analysis, interpretation of data, the writing of this article or the decision to submit it for publication.

## Conflict of Interest

The authors declare that the research was conducted in the absence of any commercial or financial relationships that could be construed as a potential conflict of interest.

## Publisher’s Note

All claims expressed in this article are solely those of the authors and do not necessarily represent those of their affiliated organizations, or those of the publisher, the editors and the reviewers. Any product that may be evaluated in this article, or claim that may be made by its manufacturer, is not guaranteed or endorsed by the publisher.

## Supplementary Material

Supplementary Figure 1 Procedure for computing Concordance Index

Supplementary Figure 2 Trees generated from XGBoost. “Gain” represents the improvement in accuracy that a feature brings to the branch on which it is on, “Cover” is the relative amount of observation concerned by a feature. While the baseline hazard h(0) is set as 0.5, the predicted hazard, h(t) is given by 0.5*exp(s) where the value s represents the summed leaf value.
