# Supplementary material for: Augmented Features Synergize Radiomics in Post-Operative Survival Prediction and Adjuvant Therapy Recommendation for Non-Small Cell Lung Cancer
Source: Front Oncol. 2022 Jan 31;12:659096. doi: 10.3389/fonc.2022.659096 (PMC8841850; doi:10.3389/fonc.2022.659096)
Supplement: Supplementary Figure 1 — Procedure for computing Concordance Index [file Presentation_1.pdf]

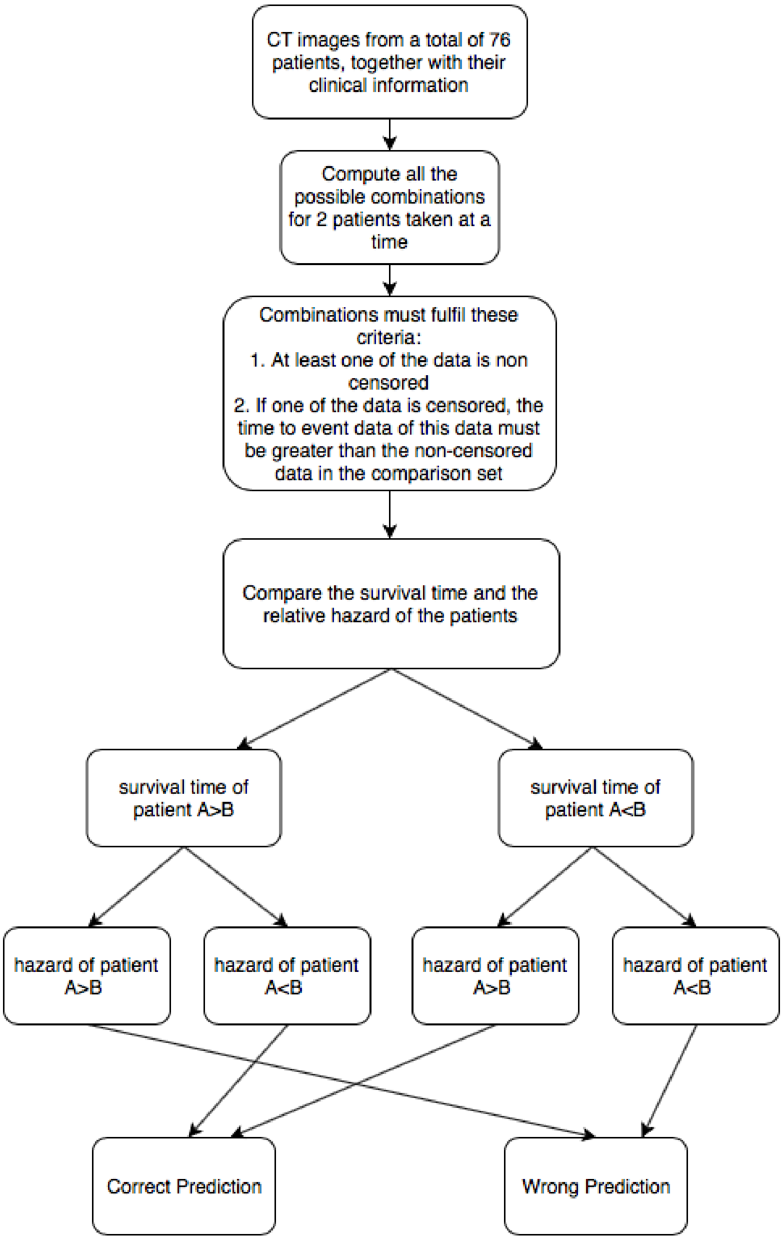

Supplementary Figure 1. Procedure for computing Concordance Index

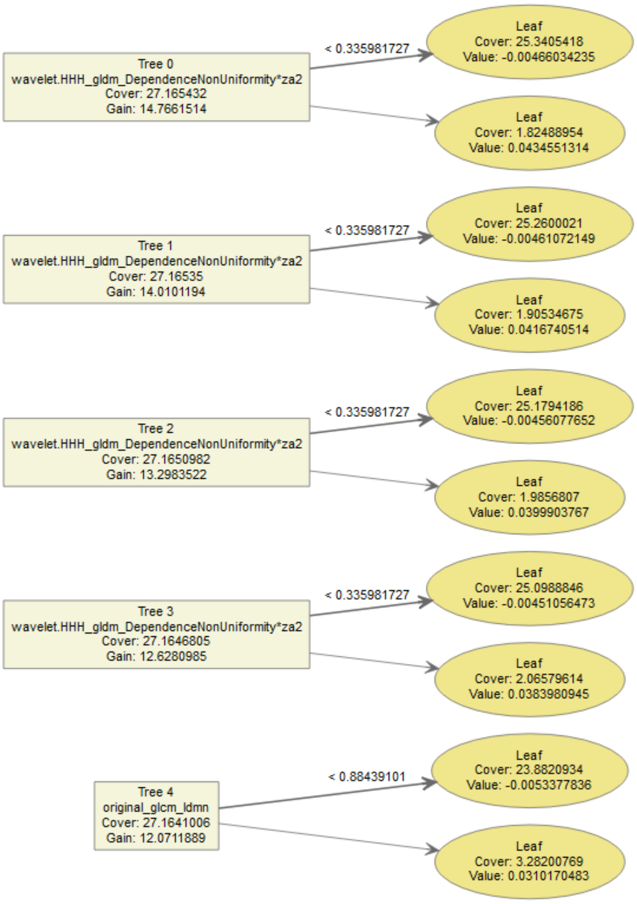

Supplementary Figure 2. Trees generated from XGBoost. “Gain” represents the improvement in accuracy that a feature brings to the branch on which it is on, “Cover” is the relative amount of observation concerned by a feature. While the baseline hazard h(0) is set as 0.5, the predicted hazard, h(t) is given by 0.5*exp(s) where the value s represents the summed leaf value.
